# Subcutaneous Emphysema Following Tracheocutaneous Fistula Closure in a Pediatric Patient: A Case Report

**DOI:** 10.7759/cureus.100974

**Published:** 2026-01-07

**Authors:** AbdulRahman R AlZayani, Rashed Aldoseri, Mohamed Alshehabi, Mai Nasser

**Affiliations:** 1 Otolaryngology - Head and Neck Surgery, Royal Medical Services - Bahrain Defence Force Hospital, Riffa, BHR; 2 Ear, Nose, and Throat (ENT), Royal Medical Services - Bahrain Defence Force Hospital, Riffa, BHR

**Keywords:** pediatric airway emergency, post op subcutaneous emphysema, subcutaneous emphysema management, tracheocutaneous fistula, tracheostomy complications

## Abstract

Tracheocutaneous fistula (TCF) is a known complication following tracheostomy decannulation. While closure is generally safe, rare but potentially life-threatening complications such as subcutaneous emphysema may occur. We report the case of a four-year-old boy with a history of prematurity, cleft palate, cleft lip, and ectopic kidney, who developed persistent TCF after tracheostomy performed for aspiration pneumonia at two months of age. Following decannulation and subsequent scheduling for elective closure, the patient underwent primary surgical repair under general anesthesia. Ten hours postoperatively, he developed diffuse subcutaneous emphysema with respiratory distress. Emergency intervention included reopening of the wound, intubation, drain placement, and re-suturing, followed by intensive care monitoring. Imaging confirmed extensive subcutaneous emphysema and a small pneumothorax. Supportive management with mechanical ventilation, nasogastric feeding, intravenous fluids, and antibiotics led to gradual resolution. He was discharged in stable condition after six days and has remained well on follow-up. Postoperative ventilation was essential to maintain airway stability despite the potential risk of propagation. There is a need for individualized perioperative planning and vigilant monitoring in pediatric patients. TCF closure is generally safe, but clinicians should remain alert to potential airway complications such as subcutaneous emphysema. Early recognition and prompt management are critical for favorable outcomes. This case emphasizes the importance of careful perioperative decision-making, especially in children with multiple comorbidities.

## Introduction

Tracheocutaneous fistula (TCF) is an abnormal communication between the trachea and the skin. It occurs as a complication of tracheostomy tube decannulation, with incidence rates varying across published studies. Historically, persistent TCF incidence was reported between 1-3% [1,2]. But more recent studies have reported incidence up to 65% [3]. It usually persists in patients who are tracheostomized for more than a year [4,5].

The closure of TCFs carries inherent risks, one of which is subcutaneous emphysema, a rare but serious complication that may require urgent surgical intervention [6]. Subcutaneous emphysema occurs when air escapes from the trachea into the subcutaneous tissue, with multiple factors contributing to the occurrence of this complication. In the context of TCF repair, it is usually due to incomplete closure of the trachea or disruption of the surgical repair, leading to air leakage into the surrounding soft tissues. Although rare and infrequently reported as a standalone complication, it is typically included within the broader rates of complications associated with TCF repair.

Multiple techniques have been described for TCF closure, most notably primary closure and secondary closure (healing by secondary intention). While both techniques demonstrate high success rates, each carries distinct theoretical advantages and risks, which are typically considered on a case-by-case basis. Studies show varying rates of complications following TCF, particularly subcutaneous emphysema, with rates ranging from 3.6%-20.4% [1,7,8]. 

We report a pediatric case of subcutaneous emphysema following TCF closure in a four-year-old male child and review the management of this complication.

## Case presentation

A four-year-old boy presented to our clinic with a persistent tracheocutaneous fistula. The patient was born prematurely and had multiple comorbidities, including cleft palate, cleft lip, and an ectopic kidney. At two months of age, he developed severe aspiration pneumonia, necessitating intubation and, eventually, tracheostomy.

Following multidisciplinary assessment, the patient underwent planned tracheostomy decannulation one year later, which was performed uneventfully. Decannulation was not surgical in nature, and the patient was discharged without immediate complications. However, the patient later returned to our hospital seeking closure of the persistent fistula. After clearance by the pediatric and anesthesia teams, the patient was scheduled for tracheocutaneous fistula closure under general anesthesia four years after the tracheostomy.

**Figure 1:**
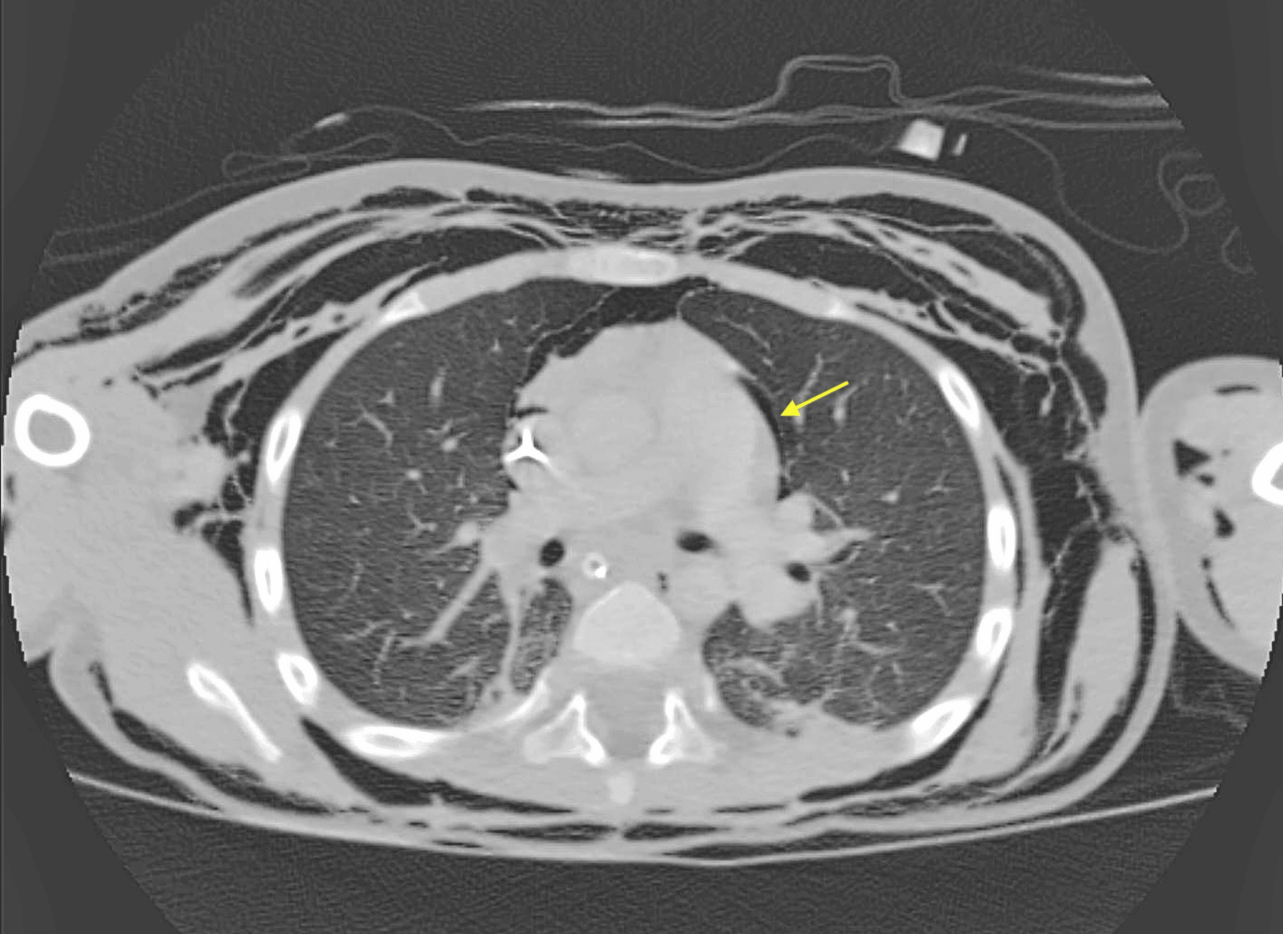
Axial CT Chest demonstrating left-sided pneumothorax (yellow arrow) This image highlights the presence of a small but clinically significant pneumothorax seen after tracheocutaneous fistula closure.

**Figure 2:**
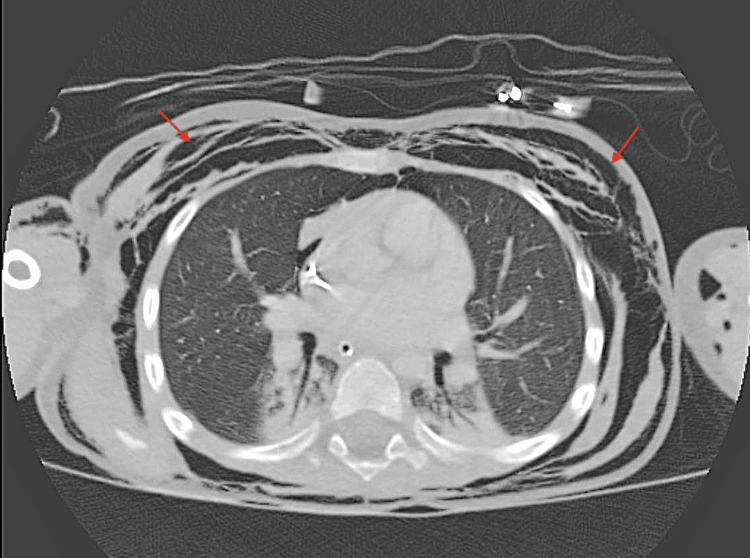
Axial CT Chest demonstrating extensive subcutaneous emphysema (red arrows) involving the anterior and lateral chest wall Air is seen dissecting through the soft tissues circumferentially around the thorax. This level demonstrates the most prominent extent of postoperative emphysema.

**Figure 3:**
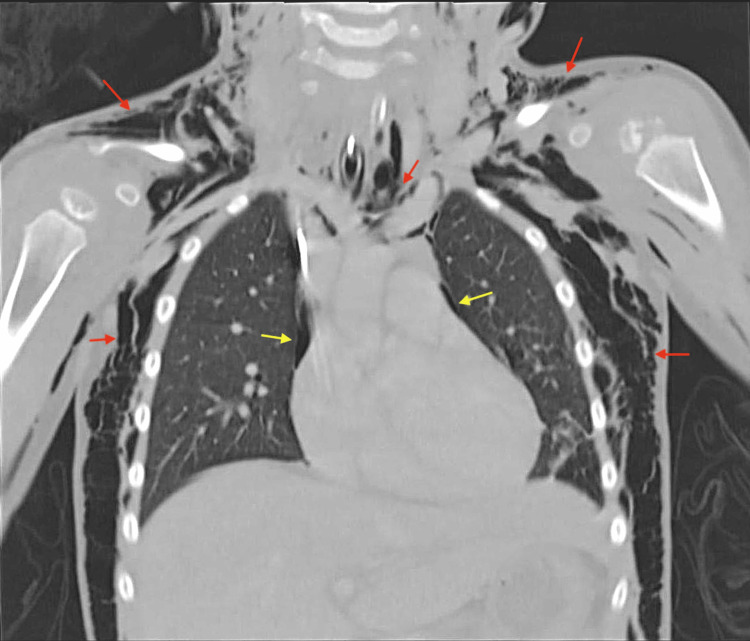
Coronal CT Chest showing diffuse surgical emphysema and bilateral small pneumothoraces Immediately postoperative CT demonstrating extensive diffuse surgical emphysema (red arrows) involving the neck, upper thorax, and bilateral upper limbs. Multiple small bilateral pneumothoraces (yellow arrows) are also visible. The image highlights the widespread air tracking through the subcutaneous and fascial planes.

Intraoperatively, direct laryngobronchoscopy was performed to confirm airway patency. A small fusiform incision was made, followed by dissection of the tracheocutaneous fistula tract down to the level of the trachea, where it was excised. The tracheal opening and overlying muscle were closed with Vicryl sutures, and the skin was closed using Monocryl sutures. The patient remained stable throughout the procedure and recovery. Ten hours postoperatively, the patient developed generalized body, neck, and facial swelling, accompanied by difficulty breathing. On examination, diffuse subcutaneous crepitus was noted, and a diagnosis of surgical emphysema was made. Emergency measures were initiated, including removal of some sutures, and the patient was immediately taken back to the operating room due to concerns about airway compromise and the potential need for emergency tracheostomy. In the operating room, intubation was successfully achieved, a drain was placed, and the surgical site was re-sutured. The patient was then transferred to the intensive care unit (ICU) for further management. A CT scan of the chest and neck revealed extensive subcutaneous emphysema and a small right pneumothorax* *(Figures 1-3).

He was placed on mechanical ventilation to secure the airway, and a nasogastric tube was inserted for feeding. Intravenous hydration and antibiotics were initiated to prevent infection. Over the next five days, the patient showed gradual improvement. Serial chest X-rays confirmed resolution of the subcutaneous emphysema. Mechanical ventilation was weaned, and the patient maintained normal oxygen saturation on room air. The nasogastric tube was removed, and the patient tolerated oral intake well. The surgical emphysema resolved completely, and the drain was removed. Six days after admission, the patient was discharged from the hospital in good condition. Subsequent outpatient follow-up visits confirmed continued recovery and stability. He was eventually discharged from our clinic in a stable condition.

## Discussion

TCF closure is a relatively routine procedure; however, it can lead to several life-threatening complications such as subcutaneous emphysema, pneumothorax, pneumomediastinum, and acute airway compromise if not recognized early and addressed promptly [6]. Pediatric patients may be particularly vulnerable due to smaller airway caliber, reduced pulmonary reserve, and a higher prevalence of congenital or syndromic conditions.

In a similar case, a four-year-old child, who had been tracheostomized for a year, presented with a persistent tracheocutaneous fistula [9]. After primary surgical closure, the patient was immediately taken back to the operating room and re-cannulated. Another case report features a case of a four-year-old boy who developed severe subcutaneous emphysema and pneumomediastinum two days after a TCF excision. This required a return to surgery, intubation, and placement of a drainage tube to release air [10]. These cases parallel ours in the need for close monitoring, appropriate precautions, and high clinical suspicion when dealing with TCF closure in the pediatric population, as many of the signs and symptoms may not be evident postoperatively.

Similar to the variability in TCF incidence, research also varies regarding complication rates, particularly subcutaneous emphysema. For example, a retrospective cohort study reported that 20.4% of patients experienced a post-TCF repair complication, 8.4% of which was subcutaneous emphysema [7]. A systematic review reports a rate of subcutaneous emphysema of 3.6-3.8% [1], while a case series reports an incidence of 18% [8].

Smith et al.'s study highlights a correlation between TCF closure and complications, specifically associated with positive airway pressure (PAP), showing 50% of all patients who received PAP developed a complication compared to 16.7% of those who did not [7]. The complications included subcutaneous emphysema, pneumomediastinum, and/or pneumothorax at a rate of 33.3% in patients receiving PAP. Despite the relatively small sample size of patients receiving PAP, the reported P-value of 0.005 highlights the significance of peri- and postoperative considerations in cases of TCF closure, and the need to monitor risk factors that may contribute to the propagation of subcutaneous emphysema in the recovery period [7]. In cases of airway compromise, it remains a critical intervention to maintain airway patency and prevent respiratory failure.

The aforementioned studies stress the importance of early recognition of complications to prevent airway compromise, which implicitly supports the notion that early management is crucial for better outcomes. Mechanical ventilation constitutes a form of PAP. Given that our patient was placed on mechanical ventilation postoperatively to secure the airway, a necessary intervention given the severity of the surgical emphysema and risk of respiratory compromise, it may have contributed to the propagation of surgical emphysema. However, it was also critical in maintaining airway patency and preventing respiratory compromise. This highlights the intricacies of the risks and benefits of PAP in the management of postoperative airway complications and further emphasizes the need for individualizing perioperative planning and vigilant monitoring postoperatively, especially in pediatric patients.

Although our patient did not have a documented diagnosis of obstructive sleep apnea (OSA) or undergo a formal sleep study, OSA is an important consideration in pediatric airway management. OSA-related upper airway obstruction and elevated intrathoracic pressures may theoretically increase the risk of postoperative air leakage and should be considered when planning TCF closure [7].

The management of TCF involves several surgical techniques with varying rates of success and complications. Two common approaches are primary and secondary closure (healing by secondary intention). The choice of technique often depends on multiple factors and is determined on a case-by-case basis by the primary team. Primary closure involves excising the fistulous tract and closing the tracheal opening in layers with sutures. Studies have reported high success rates with primary closure, although complications such as subcutaneous emphysema, pneumothorax, and wound dehiscence can occur [1,6]. Secondary closure or healing by secondary intention allows the wound to heal gradually without suturing the tracheal defect, minimizing the risk of tension on the surgical site, theoretically reducing tension and minimizing the risk of air entrapment.

A systematic review compared the rates of complications in primary vs. secondary closure, namely emphysema, and despite the theoretical differences, found no major difference between the techniques, with both reporting rates of 3.8% [1]. According to another study, the overall major complication rate in primary closure was 3.4%, with a slightly higher rate than secondary closure. The results from these studies suggest that there is minimal difference in risk [11]. In our case, primary closure was the method chosen for the procedure, given that it is the most common and well-studied method of TCF closure. The evidence showcasing no difference in the rate of complications also guided our decision towards primary closure.

In retrospect, prophylactic drain placement may have been beneficial in our patient, particularly given his multiple comorbidities and syndromic features. It is important to mention the fact that the patient had multiple comorbidities as a cleft palate and an ectopic kidney, which could affect the surgical outcome and may have played a part in the development of complications. Drain placement during the initial procedure may reduce the risk of postoperative air accumulation and should be considered in high-risk pediatric patients. 

The limited reporting of subcutaneous emphysema as a separate complication of TCF closure hinders the comparison of our case to other studies. Larger studies need to be conducted to corroborate the findings with more statistically relevant data and compare different surgical techniques.

## Conclusions

TCF closure is generally a safe and effective procedure, but complications such as subcutaneous emphysema can occur, especially in pediatric patients with complex medical histories. This case highlights the importance of early recognition and prompt management of subcutaneous emphysema to prevent airway compromise and further complications. While primary closure remains the most common method, careful patient selection and close postoperative monitoring are crucial for successful outcomes. Future studies are needed to evaluate long-term outcomes and to compare the efficacy of different surgical techniques in reducing the rate of complications like subcutaneous emphysema.
